# Retinal parameter analysis and diagnostic potential exploration in familial exudative vitreoretinopathy using ultra-widefield fundus photography

**DOI:** 10.1186/s40942-025-00716-y

**Published:** 2025-07-30

**Authors:** Jiayu Li, Shaochi Zhang, Xiaolong Qi, Chanjuan Wang, Wen Zhang, Rui Li, Caihong Sun, Keyan Liu, Xiaolu Li, Wenjuan Zhuang

**Affiliations:** 1 Department of Ophthalmology, People’s Hospital of Ningxia Hui Autonomous Region (Affiliated Hospital of Ningxia Medical University), Zhengyuan Road, Yinchuan, Ningxia, 750011 China; 2 Third Clinical Medical College of Ningxia Medical University, Zhengyuan Road, Yinchuan, Ningxia 750011 China

**Keywords:** Familial exudative vitreoretinopathy, Ultra-widefield fundus photography, Retinal parameters

## Abstract

**Background:**

Familial Exudative Vitreoretinopathy (FEVR) is a monogenic disorder causing retinal vascular impairment, often underdiagnosed due to its variable presentation and reliance on invasive methods like fundus fluorescein angiography (FFA). Through the utilization of non-invasive ultra-widefield fundus photography (UWFFP), this research explored both the diagnostic potential of integrated retinal parameters for the detection of FEVR, and their characteristic changes during the early stage progression.

**Methods:**

Retinal parameters were systematically extracted and quantified from UWFFP of 114 FEVR patients and 114 matched controls using the EVision AI cloud platform. Comparative statistical analyses were performed to identify significant intergroup differences between FEVR and control cohorts, and assess intra-group variations among FEVR subgroups. Based on parameters that showed significant differences between the FEVR group and the control group and had an impact on the FEVR group, a diagnostic model was constructed. Receiver operating characteristic (ROC) curves were plotted to determine the diagnostic potential of these parameters. In addition, subgroup analysis within the FEVR group was conducted to clarify the relationship between retinal parameters and disease staging.

**Results:**

Significant differences were observed in 25 retinal parameters between the FEVR group and the control group, with the horizontal cup-to-disc ratio, vertical cup-to-disc ratio, optic disc-to-macula distance, and vascular density demonstrating potential diagnostic efficacy. Subgroup analysis within the FEVR group revealed that as the disease stage advanced and severity increased, the optic disc and cup diameters decreased, the optic disc-to-macula distance increased, and the vascular fractal dimension and vascular density parameters declined.

**Conclusions:**

UWFFP and automated retinal parameter analysis offer promising tools for early FEVR diagnosis, with specific structural and vascular markers providing diagnostic potential. Further large-scale studies are needed to validate these findings and refine diagnostic models.

**Supplementary Information:**

The online version contains supplementary material available at 10.1186/s40942-025-00716-y.

## Introduction

Familial Exudative Vitreoretinopathy (FEVR) is a monogenic disorder characterized by impaired retinal vascular development, primarily manifesting as incomplete vascularization, resulting in peripheral retinal avascularity and hypoxia [1, 2]. Clinical features include peripheral retinal avascular zones [3], increased vascular branching with tortuosity, retinal neovascularization, leakage, peripheral fibrosis, vitreous hemorrhage, persistent fetal vasculature, vitreoretinal traction, macular displacement, retinal folds, and retinal detachment [4]. FEVR exhibits incomplete penetrance with a broad spectrum of severity, ranging from asymptomatic cases to congenital blindness or progressive vision loss due to complications such as vitreous hemorrhage and retinal detachment [5]. Recent neonatal retinal screening and wide-angle fundus imaging studies indicate a prevalence of 0.66–1.19% in China, suggesting FEVR is more common than previously recognized.

Diagnosing FEVR presents significant clinical challenges due to its phenotypic variability and incomplete penetrance. Current diagnostic approaches typically require a comprehensive evaluation incorporating family history assessment, genetic testing, and fundus fluorescein angiography (FFA) [6, 7]. FEVR frequently evades timely diagnosis due to its phenotypic overlap with other retinal disorders, notably high myopia and retinopathy of prematurity (ROP), particularly in pediatric populations or when FFA is clinically contraindicated. Compounding this diagnostic challenge is the limited disease awareness among primary care providers and general ophthalmologists [5, 8]. Consequently, researchers have proposed that if FEVR could be accurately diagnosed without the need for FFA, it would significantly streamline clinical diagnosis and management [9].

Recent studies have explored retinal microstructural and vascular parameters using ultra-widefield fundus photography (UWFFP), offering a novel approach for diagnosis and early intervention [10, 11]. UWFFP is widely utilized in clinical and pediatric retinal screening due to its non-invasive nature, ability to image peripheral retina without pupil dilation or contrast agents, and capacity to capture avascular zones, abnormal vascular branching, neovascularization, and lattice degeneration. Previous investigations have primarily concentrated on the scrutiny of retinal parameters within restricted or single - modal contexts. Building on this fundamental research, the present study expands the analytical framework by employing an artificial intelligence (AI)-powered platform to automatically segment and extract parametric features of the optic disc, optic cup, macula, and retinal microvasculature. This methodology aims to leverage UWFFP and AI-assisted non-invasive techniques to explore the potential for early detection of FEVR, and facilitates timely monitoring of disease progression, enabling proactive intervention for this vision-threatening condition.

## Methods

### Study population

Between September 2021 and December 2023, 213 patients diagnosed with FEVR at the Ophthalmology Department of Ningxia Hui Autonomous Region People’s Hospital were first selected. The gender, age, birth history, oxygen therapy history, family history, and treatment history of FEVR probands and their family members were recorded. The affected eyes, and collect best-corrected visual acuity (BCVA), intraocular pressure, ocular examination, as well as imaging data from UWFFP (Daytona P200T, OPTOS, Fife, UK) and FFA (Spectralis HRA + OCT, Heidelberg Engineering GmbH, Heidelberg, DE) were clearly identified. The disease stage in patients with FEVR was determined for each eye using Trese’s staging system based on FFA findings. All participants provided written informed consent. The study complied with the Declaration of Helsinki and approved by the Ethics Committee of Ningxia Hui Autonomous Region People’s Hospital (No. 2020-KY-GZR019).

### Inclusion and exclusion criteria

#### The inclusion criteria for the FEVR group were as follows

The diagnosis of FEVR is confirmed through a positive family history or genetic evidence, fundus examination, FFA, and UWFFP. Based on the diagnostic criteria proposed by Kashani et al. [6, 7], early stage FEVR should be suspected when FFA demonstrates characteristic findings including abnormally increased retinal vascular branching with dense clustering, fan-shaped termination of vessels at the equatorial region accompanied by anastomotic loops at vascular endpoints, well-demarcated peripheral capillary non-perfusion zones extending > 2 disc diameters from the ora serrata, and/or abnormal fluorescein leakage at vascular termini or anastomotic sites. In UWFFP images, the entire retinal imaging area, encompassing the optic disc, macular region, and vortex veins, must be clearly captured, with the retinal vasculature being distinctly visualized. The images should be free from iris shadowing, lens cap obstruction, or excessive eyelid/eyelash coverage (≤ 10° of the retinal imaging area). Additionally, normal intraocular pressure (ranging from 10 to 21 mmHg) and clear optical media were required.

#### The exclusion criteria for the FEVR group were as follows

History of prematurity, oxygen therapy, vascular ocular diseases, glaucoma, systemic disorders, or related syndromes; Coexisting vitreoretinal pathologies (e.g., Norrie disease, persistent fetal vasculature, macular holes, retinal hemorrhage, or detachment); Active ocular inflammation, trauma, or prior ocular surgery; Poor-quality images or ocular conditions causing compromised visualization of the optic disc, macula, or vasculature, or insufficient imaging range; Recent use (within 2 weeks) of medications affecting vascular function. Notably, syndromic FEVR cases such as Knobloch syndrome were specifically excluded because their additional systemic manifestations and more severe ocular comorbidities could confound the analysis of isolated FEVR retinal changes. This approach enabled the establishment of well-defined baseline parameters for idiopathic FEVR while ensuring comparability with existing literature. After exclusions, 114 eligible subjects were stratified into FEVR groups based on FFA and UWFFP, the eye with the highest stage of the disease was selected as the study eye. (Fig. S1).

#### The inclusion and exclusion criteria for the control group were as follows

The control group comprised 114 myopic patients (spherical equivalent − 1.00 to -6.00 D) matched to FEVR cases using propensity score matching (1:1 nearest-neighbor method, caliper width 0.2 standard deviations). Matching criteria included axial length (± 0.5 mm), age (± 2 years), gender (exact match), and spherical equivalent (± 1.00 D). Axial length received priority in the matching algorithm based on established correlations with ocular parameters. All control subjects met UWFFP image quality requirements and showed no evidence of ocular or systemic comorbidities.

### Retinal parameter extraction and quantification

Optos UWFFP images were analyzed using the automated EVision AI Cloud Platform (EVision Technology Co., Ltd., Beijing, China). This software integrated computer vision and deep learning technologies to enhance edge recognition of key anatomical features. By combining deep object detection networks with edge extraction algorithms, it achieved subpixel-level segmentation of the optic disc, optic cup, and macula. For vascular networks, the ResNet101-UNet segmentation architecture classified arteries and veins based on chromatic characteristics, luminance distribution, textural patterns, and topological connections of blood vessels. Validation on an independent test set demonstrated segmentation accuracy ≥ 96%, sensitivity ≥ 85%, and specificity ≥ 96% for the optic disc, optic cup, macula, and vascular structures [12, 13]. Image quality was assessed using predefined automated segmentation criteria, evaluating exposure adequacy and accurate identification of the optic disc and macula. Images that met quality thresholds underwent automated segmentation and quantification of retinal parameters (Fig. S2). To ensure comparability, the region of interest (ROI) pixel area in control group UWFFP images was matched to the FEVR group, with no statistically significant intergroup differences, enabling subsequent analysis of retinal and vascular parameters. A total of 36 retinal parameters, including optic disc, optic cup, macula, and retinal vasculature metrics, were compared between the FEVR and control groups.

### Statistical analysis

Statistical analysis was performed using SPSS 26.0 (IBM, USA). Mann-Whitney U tests and Kruskal-Wallis tests were used for data not conforming to a normal distribution, while independent sample t-tests and ANOVA were used for normally distributed data. Spearman’s rank correlation analysis was applied to examine the relationship between retinal parameters and group differences. To mitigate multicollinearity identified through correlation and variance inflation factors (VIF) analysis, we applied forward selection logistic regression (*P* < 0.05 for entry), which iteratively incorporates only the most significant predictor, thus preventing highly correlated variables from co-entering the model [14]. Diagnostic models were subsequently constructed to evaluate the diagnostic utility of retinal parameters, with receiver operating characteristic (ROC) curves generated from the regression outputs. Statistical significance was defined as *P* < 0.05.

## Results

### Demographic and axial length characteristics between the FEVR and control groups

Following quality control and screening, 114 FEVR patients (114 eyes), including a subset with genetically confirmed diagnoses (Supplementary Table 1), and 114 matched control subjects (114 eyes) were enrolled for retinal parameter analysis. Demographic characteristics including sex distribution (FEVR: 63 male/51 female vs. control: 61 male/53 female), mean age (15.36 ± 0.83 vs. 15.23 ± 0.90 years), and axial length (24.75 ± 2.35 vs. 24.43 ± 2.01 mm) showed no statistically significant differences between groups (all *P* > 0.05), ensuring comparable baseline characteristics for subsequent parameter comparisons. (Table 1).

**Table 1:** Demographic and axial length characteristics of the FEVR and control groups

Variable	FEVR (*n* = 114)	Control (*n* = 114)	*P*-value
Sex (Male/Female)	63/51	61/53	0.79
Age (years)	15.36 ± 0.83	15.23 ± 0.90	0.258
Axial Length (mm)	24.75 ± 2.35	24.43 ± 2.01	0.27

### Retinal parameter analysis in FEVR patients

#### Comparison of retinal parameters between the FEVR and control groups

According to Table 2, there were no differences in ROI parameters between the FEVR and control groups, allowing for further analysis. A total of 25 parameters (the definitions and research significance of these 25 parameters can be found in Supplementary Table 2.) related to the optic disc, macula, and vascular structures were found to be significantly different between the two groups (*P* < 0.05). The 11 parameters (ROI, optic disc tilt angle, optic disc long axis angle to vertical line, maximum disc-cup rim distance, macula-optic disc line angle to optic disc long axis, optic disc vertical-to-horizontal axis ratio, average vessel tortuosity, optic disc vertical diameter, Optic disc area, optic disc elliptical long axis, optic disc radius) showed no significant intergroup differences (*P* > 0.05).

**Table 2:** Comparison of parameters between the FEVR group and the control group

Retinal parameters (Unit)	Group		*P*-value
	**FEVR **(***n* = 114)**	**Control **(***n* = 114)**	
ROI^a^	9039661.000	9370361.500	0.806
Optic cup horizontal diameter (µm) ^a^	924.000	1162.000	0.000**
Optic cup area (µm²) ^a^	662676.000	1058105.000	0.000**
Minimum disc-cup rim angle to horizontal line - end (°) ^a^	-35.020	-15.255	0.003**
Minimum disc-cup rim angle to horizontal line - start (°) ^a^	-35.391	-15.069	0.002**
Rim S distance (µm) ^a^	581.000	476.000	0.000**
Rim N distance (µm) ^a^	525.000	462.000	0.000**
Rim T distance (µm) ^a^	497.000	448.000	0.000**
Optic disc-to-macula distance (µm) ^a^	6727.935	5977.830	0.000**
Macula-optic disc line angle (°) ^a^	7.316	9.884	0.002**
Optic disc short-to-long axis ratio ^a^	0.827	0.879	0.000**
Optic disc long-to-short axis ratio ^a^	1.210	1.138	0.000**
Optic cup roundness ^a^	0.775	0.831	0.000**
Vascular density ^a^	0.036	0.046	0.000**
Average vessel diameter (µm) ^a^	106.364	108.065	0.000**
Vessel length (mm) ^a^	635442.735	734158.369	0.000**
Vessel fractal dimension ^a^	1.424	1.441	0.000**
Optic cup vertical diameter (µm) ^b^	967.84 ± 239.41	1166.05 ± 226.92	0.000**
Optic disc horizontal diameter (µm) ^b^	2012.30 ± 262.01	2095.82 ± 247.38	0.01*
Optic disc elliptical short axis (µm) ^b^	1886.44 ± 276.19	1982.47 ± 232.56	0.005**
Rim I distance (µm) ^b^	516.28 ± 121.27	486.56 ± 98.37	0.043*
Minimum disc-cup rim distance (µm) ^b^	385.74 ± 94.23	345.19 ± 87.42	0.001**
Area ratio of cup-disc ^b^	0.21 ± 0.09	0.31 ± 0.08	0.000**
Horizontal cup-disc ratio ^b^	0.45 ± 0.10	0.57 ± 0.07	0.000**
Vertical cup-disc ratio ^b^	0.45 ± 0.10	0.54 ± 0.07	0.000**
Optic disc roundness ^b^	0.78 ± 0.09	0.83 ± 0.07	0.000**

Explanations regarding the units, symbols, abbreviations, and statistical parameters presented in the table: µm: micrometers; mm: millimeters; °: degrees; Parameters with *P* > 0.05 are not shown in the table; Parameters with superscript “a” are analyzed using the Mann-Whitney U test; parameters with superscript “b” are analyzed using the T-test; parameters showing statistically differences (*P* < 0.05) in two groups are indicated in bold; “*” denotes *P* < 0.05, “**” denotes *P* < 0.01

#### Correlation analysis between FEVR group and retinal parameters

**Fig. 1:**
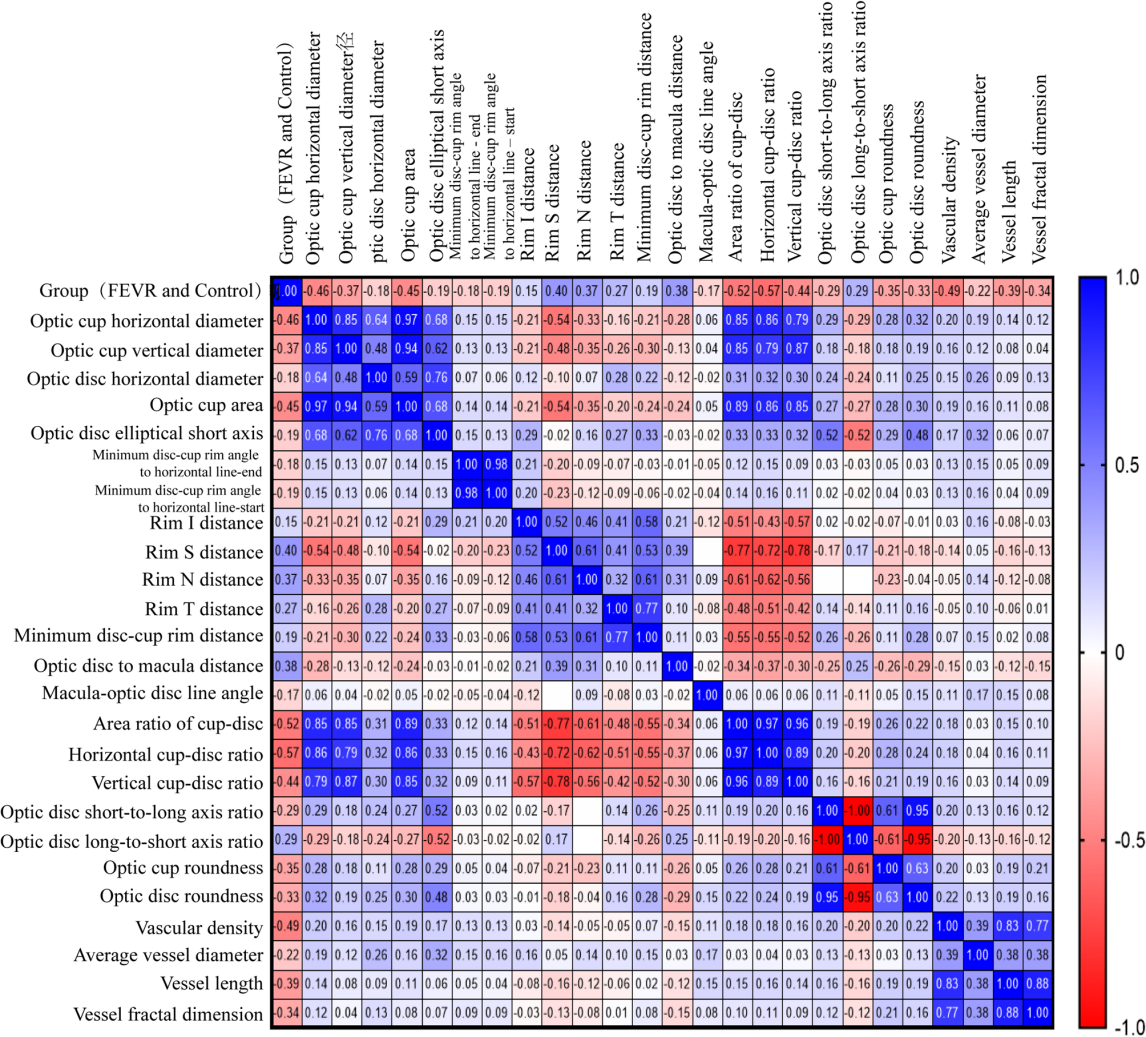
Spearman correlation analysis between group (FEVR and control) and retinal Parameters, and between retinal parameters. The x-axis and y-axis represent the 25 parameters with significant differences between the FEVR and control groups. A correlation closer to + 1 or -1 indicates a stronger relationship

Spearman’s correlation coefficient (r) was used to assess the strength of the correlation. The analysis revealed that the rim S distance (*r* = 0.431, *P* < 0.01) and the optic disc-to-macula distance (*r* = 0.430, *P* < 0.01) were significantly positively correlated with the group, indicating that these parameters were higher in the FEVR group compared to the control group. Conversely, the optic cup horizontal diameter (*r*=-0.473, *P* < 0.01), optic cup area (*r*=-0.459, *P* < 0.01), Area ratio of cup-disc (*r*=-0.543, *P* < 0.01), horizontal cup-disc ratio (*r*=-0.580, *P* < 0.01), vertical cup-disc ratio (*r*=-0.460, *P* < 0.01), vascular density (VD) (*r*=-0.537, *P* < 0.01), vascular length (VL) (*r*=-0.441, *P* < 0.01), and vascular fractal dimension (VFD) (*r*=-0.398, *P* < 0.01) were significantly negatively correlated with the group, with these parameters being smaller in the FEVR group compared to the control group (Fig. 1). Additionally, correlations were observed between the optic disc-cup parameters and vascular parameters (Fig. 1).

#### Diagnostic performance of retinal parameters in FEVR patients

**Fig. 2:**
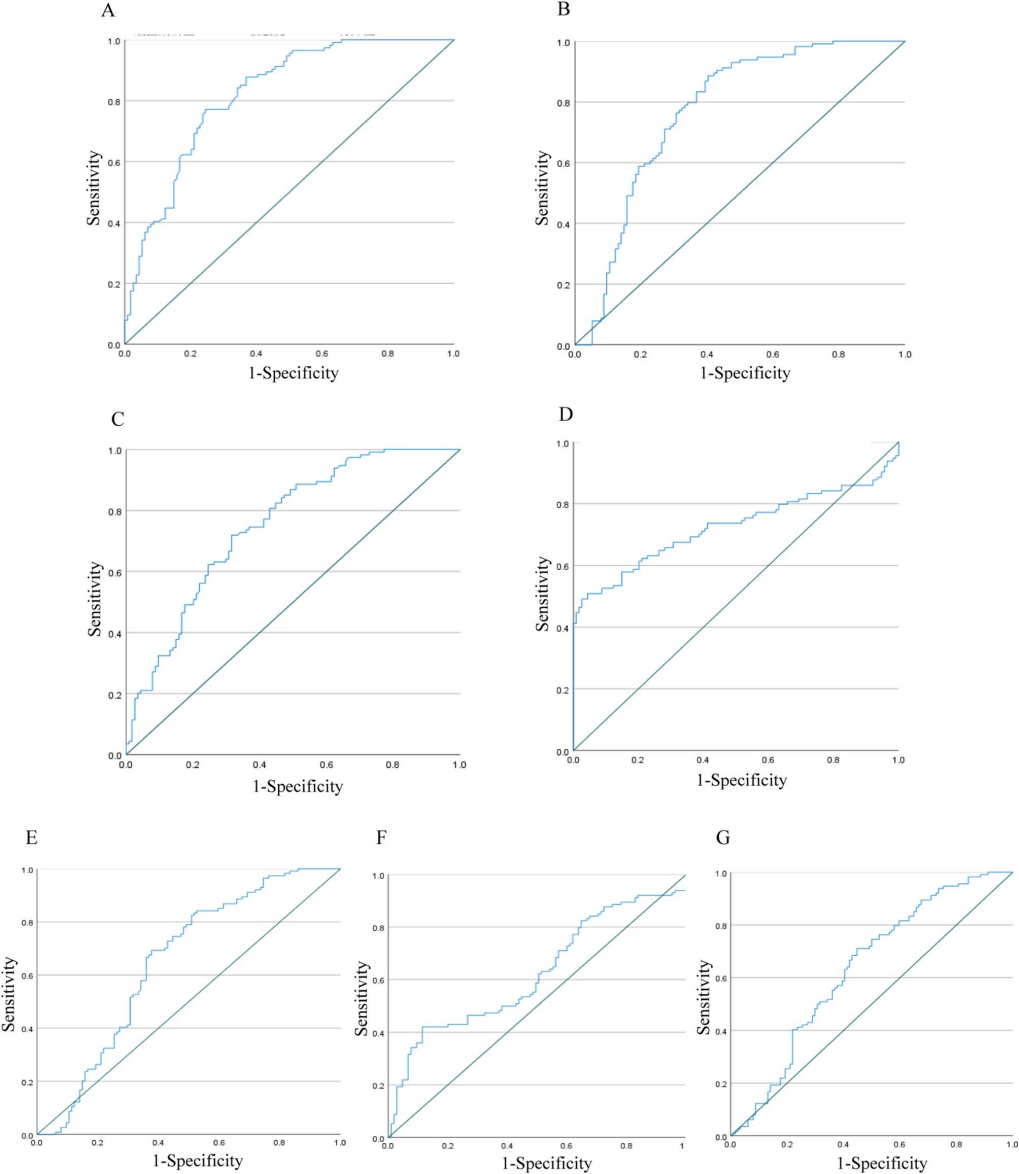
ROC curves of retinal parameters, representing their sensitivity and specificity. **A** Horizontal cup-disc ratio. **B** Vascular density. **C**. Vertical cup-disc ratio. **D** Optic disc-to-macula distance. **E** Vascular fractal dimension. **F** Macula-optic disc line angle. **G** Average vessel diameter

Based on the correlation analysis results between the FEVR and control groups, parameters were selected for logistic regression (Table 3). Consistent with TRIPOD guidelines for prediction models [15], clinically critical variables (e.g., horizontal and vertical cup-to-disc ratios) were retained despite moderate collinearity (VIF < 10) to preserve essential diagnostic information. Ultimately, seven parameters include optic disc-to-macula distance, macula-optic disc line angle, VD, average vessel diameter (AVD), FD, horizontal cup-disc ratio, and vertical cup-disc ratio remained in the model. These parameters were found to have a causal relationship with FEVR. ROC curve analysis revealed that horizontal cup-disc ratio (AUC = 0.822, 95% CI: 0.768–0.876), VD (AUC = 0.77, 95% CI: 0.707–0.834), vertical cup-disc ratio (AUC = 0.753, 95% CI: 0.69–0.815), and optic disc-to-macula distance (AUC = 0.725, 95% CI: 0.655–0.794), showed moderate-to-good performance in predicting FEVR (AUC > 0.7, *P* < 0.05) (Fig. 2), whereas vascular fractal dimension (AUC = 0.651, 95% CI: 0.578–0.724), macula-optic disc line angle (AUC = 0.624, 95% CI: 0.550–0.698), and average vessel diameter (AUC = 0.636, 95% CI: 0.563–0.708) showed relatively weaker predictive efficacy.

**Table 3:** Binary logistic regression analysis results for FEVR group and control group

Retinal parameters (Unit)	*P*-value	OR-value	95% CI
Optic disc-to-macula distance (µm)	0.001	1.001	1.000–1.002
Macula-optic disc line angle (°)	0.009	0.994	0.990–0.999
**Vascular density**	**0.000**	0.0004	0.000–0.042
Average vessel diameter (µm)	0.010	0.846	0.745–0.961
Vascular fractal dimension	0.002	0.884	0.818–0.956
**Horizontal cup-disc ratio**	**0.000**	0.0002	0.000–0.032
Vertical cup-disc ratio	0.003	0.847	0.729–0.984

Explanations regarding the units, symbols, abbreviations, and statistical parameters presented in the table: µm: micrometers; °: degrees; parameters showing statistically differences (*P* < 0.001) in two groups are indicated in bold

#### Comparison of retinal parameters among FEVR stage 1, stage 2, and control groups

The optic cup horizontal and vertical diameters, Area ratio of cup-disc, horizontal cup-disc ratio, and vertical cup-disc ratio in Stage 1 and Stage 2 of the FEVR group were smaller than in the control group (*P* < 0.05), with these parameters progressively decreasing as the disease advanced. The optic disc horizontal diameter showed a decreasing trend in both Stage 1 and Stage 2 compared to the control group, with significant differences observed between Stage 2 and the control group (*P* < 0.05). The optic disc-to-macula distance was larger in Stage 2 than in Stage 1 and the control group (*P* < 0.05), with no significant difference between Stage 1 and the control group (*P* > 0.05).

Regarding vascular parameters, Stage 2 patients had significantly lower VD and length than both the control group and Stage 1 (*P* < 0.05), with AVD smaller than in the control group (*P* < 0.05). The VFD was highest in Stage 1 of the FEVR group, even exceeding that of the control group, but significantly decreased with disease progression. In Stage 2, the VFD was significantly lower than in both Stage 1 and the control group (*P* < 0.05) (Tables 4 and 5).

Notably, Stage 2 FEVR exhibited markedly reduced vascular density (VD: 0.035 vs. 0.044, *P* < 0.001) and vessel fractal dimension (VFD: 1.411 vs. 1.456, *P* < 0.001) compared to Stage 1, along with decreased horizontal cup-disc ratio (0.44 vs. 0.49, *P* < 0.005) and increased optic disc-to-macula distance (7230.8 μm vs. 6081.1 μm, *P* < 0.009).

**Table 4:** Pairwise comparison of parameters among stage 1 FEVR, stage 2 FEVR, and control groups (Kruskal-Wallis test and bonferroni Correction)

Retinal parameters (Unit)	Group(Md)			*P*-value^a^	*P*-value^b^		
	Stage 1 FEVR(*n* = 30)	Stage 2 FEVR(*n* = 84)	Control (*n* = 114)		Stage 1 FEVR vs. Control	Stage 2 FEVR vs. Control	Stage 1 FEVR VS Stage 2 FEVR
ROI	8923647.500	9057148.500	9370361.500	0.581	1	1	0.934
Optic cup horizontal diameter (µm)	980.000	924.000	1162.000	0.000**	0.003**	0.000**	0.345
Optic cup area (µm²)	761264.000	645918.000	1058105.000	0.000**	0.003**	0.000**	0.454
Minimum disc-cup rim angle to horizontal line - end (°)	-30.537	-45.00	-15.255	0.008**	0.524	0.007**	1
Minimum disc-cup rim angle to horizontal line - start (°)	-29.400	-45.000	-15.069	0.006**	0.694	0.004**	0.903
Rim S distance (µm)	539.000	616.000	476.000	0.000**	0.052	0.000**	0.11
Rim N distance (µm)	469.000	560.000	462.000	0.000**	1	0.000**	0.002**
Rim T distance (µm)	490.000	511.000	448.000	0.000**	0.075	0.000**	1
Optic disc-to-macula distance (µm)	6081.085	7230.765	5977.830	0.000**	0.39	0.000**	0.009**
Macula-optic disc line angle (°)	3.769	8.543	9.884	0.002**	0.003**	0.08	0.333
Optic disc short-to-long axis ratio	0.835	0.824	0.879	0.000**	0.034*	0.000**	1
Optic disc long-to-short axis ratio	1.198	1.214	1.138	0.000**	0.034*	0.000**	1
Optic cup roundness	0.840	0.770	0.831	0.000**	1	0.000**	0.003**
Vascular density	0.044	0.035	0.046	0.000**	0.86	0.000**	0.000**
Average vessel diameter (µm)	106.453	106.310	108.065	0.002**	0.135	0.002**	1
Vessel length (mm)	743887.492	620513.251	734158.369	0.000**	1	0.000**	0.000**
**Vascular fractal dimension**	1.456	1.411	1.441	0.000**	0.004**	0.000**	0.000**

Explanations regarding the units, symbols, abbreviations, and statistical parameters presented in the table: µm: micrometers; mm: millimeters; °: degrees; md: median; parameters with *P* > 0.05 are not shown in the table, P-values with statistically significant differences are indicated in bold; parameters showing statistically differences (*P* < 0.05) across all three groups are indicated in bold; “*” denotes *P* < 0.05, “**” denotes *P* < 0.01; P-value^a^ represents the P-value for the overall comparison among stage 1 FEVR, stage 2 FEVR, and control groups; P-value^b^ represents the P-value for pairwise comparisons between stage 1 FEVR, stage 2 FEVR, and control groups

**Table 5:** Pairwise comparison of parameters between stage 1 FEVR, stage 2 FEVR, and control groups (ANOVA and tamhane’s T2 Correction)

Retinal parameters (Unit)	Group(-x ± s)			*P*-value^a^	*P*-value^b^		
	Stage 1 FEVR(*n* = 30)	Stage 2 FEVR(*n* = 84)	Control (*n* = 114)		Stage 1 FEVR VS Control	Stage 2 FEVR VS Control	Stage 1 FEVR VS Stage 2 FEVR
Optic cup vertical diameter (µm)	1010.80 ± 201.15	952.50 ± 262.63	1166.05 ± 226.92	0.000**	0.005**	0.000**	0.75
Optic disc horizontal diameter (µm)	2058.92 ± 115.47	1995.66 ± 268.90	2095.82 ± 247.38	0.001*	0.561	0.024*	0.227
Optic disc elliptical short axis (µm)	1855.93 ± 259.11	1897.33 ± 282.74	1982.47 ± 232.56	0.014*	0.050*	0.064	1
Rim I distance (µm)	486.27 ± 102.35	527.00 ± 126.17	486.56 ± 98.37	0.029*	1	0.034*	0.248
Minimum disc-cup rim distance (µm)	371.23 ± 72.36	390.98 ± 100.85	345.19 ± 87.42	0.002**	0.492	0.002**	0.926
**Area ratio of cup-disc**	0.25 ± 0.08	0.20 ± 0.09	0.31 ± 0.08	0.000**	0.000**	0.000**	0.021*
**Horizontal cup-disc ratio**	0.49 ± 0.08	0.44 ± 0.10	0.57 ± 0.07	0.000**	0.000**	0.000**	0.005**
**Vertical cup-disc ratio**	0.49 ± 0.09	0.44 ± 0.10	0.54 ± 0.07	0.000**	0.015*	0.000**	0.024*
Optic disc roundness	0.80 ± 0.07	0.77 ± 0.10	0.83 ± 0.07	0.000**	0.062	0.000**	0.311

Explanations regarding the units, symbols, abbreviations, and statistical parameters presented in the table: µm: micrometers; mm: millimeters; °: degrees; -x ± s: mean ± standard deviation; Parameters with *P* > 0.05 are not shown in the table, P-values with statistically significant differences are indicated in bold; parameters showing statistically differences (*P* < 0.05) across all three groups are indicated in bold; “*” denotes *P* < 0.05, “**” denotes *P* < 0.01; P-value^a^ represents the P-value for the overall comparison among Stage 1 FEVR, Stage 2 FEVR, and Control groups; P-value^b^ represents the P-value for pairwise comparisons between Stage 1 FEVR, Stage 2 FEVR, and Control groups

## Discussion

In recent years, with increasing attention on FEVR, studies have revealed that the disease is not as rare as previously thought, yet its diagnostic rate often falls short of expectations [5]. The primary reason for underestimating its incidence is that early-stage patients often present with very mild or even no symptoms at all [16]. Furthermore, the disease can progress throughout life, potentially leading to severe complications such as retinal detachment and vitreous hemorrhage [16]. Therefore, Early diagnosis of FEVR before symptomatic complications arise is critical, as delayed detection is associated with irreversible vision loss from retinal detachment, vitreous hemorrhage, or macular complications [5, 16]. Non-invasive UWFFP coupled with automated parameter analysis offers a practical tool for identifying pre-symptomatic FEVR, particularly in pediatric populations where FFA is contraindicated or poorly tolerated [9, 10]. This approach may enable earlier intervention than routine clinical exams reliant on overt structural changes. In this study, we utilize UWFFP and automated AI analysis to compare retinal parameters between the FEVR and control groups, and to explore the differences in retinal parameters within the FEVR subgroups. We further identified retinal parameters with diagnostic potential for FEVR and determine stage-dependent biomarkers for disease progression monitoring.

In our analysis of retinal parameters between the FEVR and control groups, we found differences in 25 parameters related to the optic disc, macula, and retinal vasculature. Combining optic cup horizontal diameter, optic cup vertical diameter, optic cup area, optic disc horizontal diameter, optic disc elliptical short axis, horizontal cup-disc ratio, vertical cup-disc ratio, optic cup roundness, and optic disc roundness, we concluded that FEVR patients exhibited smaller optic disc and optic cup diameters, reduced cup-disc ratios, and a decrease in optic disc roundness, along with shape changes compared to the control group. Additionally, parameters such as macula-optic disc line angle and optic disc-to-macula distance suggested that macular position shifted in FEVR patients, with increased distance between the optic disc and macula.

In studies analyzing retinal structure and vascular parameters, Boonstra et al. [5] were the first to report a strong correlation between optic disc size and optic disc-to-macula distance with the presence of an avascular zone, stating that in FEVR patients with peripheral retinal avascular zones, the optic disc tends to be smaller and the optic disc-to-macula distance longer. They suggested that these microstructural changes in the posterior pole could serve as additional markers for FEVR, providing clues for its diagnosis. Another study had also observed abnormalities in the posterior region of mildly asymptomatic FEVR patients, such as changes in vascular structure and increased distance from the macular fovea to the optic disc [17]. Yuan et al. [18] found that asymptomatic FEVR patients exhibited various abnormalities in the posterior pole, including increased retinal vessels, significantly increased optic disc-to-macula distance, and a notably smaller optic disc horizontal diameter. Ozdek et al. [9] also reported that even asymptomatic family members of FEVR patients could exhibit severe retinal peripheral vascular abnormalities, smaller optic disc diameters, and macular displacement. Our findings are consistent with Boonstra et al. [5] showing that FEVR patients have smaller optic disc horizontal and vertical diameters compared to the control group, while Yuan et al. [18] found no significant difference in the vertical diameter between the groups. The exact cause of smaller optic disc diameter in FEVR patients remains unclear. A recent study [19] suggested that vitreoretinal traction may elevate the optic disc, disrupting its nutrition and leading to optic disc hypoplasia, while peripheral retinal ischemia may be an indirect cause of smaller optic disc diameter in asymptomatic patients. Our conclusions regarding optic disc-to-macula distance and macular position change align with studies by Boonstra [5], Ozdek [9], Liu [15], and Yuan [18], which may be due to vitreoretinal interface abnormalities on the temporal side of the retina pulling the macula.

In the analysis of vascular parameters, Li et al. [20] found that in mildly affected FEVR children, retinal VD, AVD, and average curvature significantly increased, while Liu et al. [21] reported that FEVR patients exhibited significantly higher VD, AVD, and curvature around the optic disc compared to controls. Of particular importance is that whereas previous findings were obtained using 45° fundus photography, our UWFFP-based study revealed that retinal VD, AVD, VL, and VFD were all smaller in FEVR patients compared to the control group, a finding consistent with Zhang et al. [22], who found similar results in the peripheral retina using wide-field optical coherence tomography angiography (WFOCTA). They also found that the vascular index (VI) was significantly lower in FEVR patients in both the superficial capillary plexus (SCP) and deep capillary plexus (DCP) compared to controls. Animal studies have shown that mice with FEVR-related gene knockouts exhibit suppressed vascular growth in both the SCP and DCP [23, 24], which may be due to impaired Wnt signaling that regulates normal retinal vascular development [25]. Impaired retinal vascular development in FEVR primarily reduces vascular branching in the SCP, creating hypoxic conditions that trigger secondary ischemic remodeling. This ischemia-driven process induces compensatory vessel thickening and narrowing, as demonstrated in NDP knockout models [23, 24]. Concurrently, the developmental defect disrupts the DCP network, particularly affecting temporal vascular branches and capillary anastomoses, ultimately resulting in progressive avascular zones. These vascular defects may worsen as retinal vascular dysplasia becomes more severe [22].

Our study explored the diagnostic potential of combined retinal microstructure and vascular parameters, such as optic disc, optic disc-to-macula distance, and others, through logistic regression and ROC curve analysis. The results showed that horizontal cup-disc ratio (AUC = 0.822, 95% CI: 0.768–0.876), VD (AUC = 0.77, 95% CI: 0.707–0.834), vertical cup-disc ratio (AUC = 0.753, 95% CI: 0.69–0.815), and optic disc-to-macula distance (AUC = 0.725, 95% CI: 0.655–0.794) showed moderate-to-good performance for FEVR occurrence. Previous study have also established diagnostic models for FEVR using parameters such as retinal avascular area, vascular angle, VFD, vascular branching, and density, achieving high diagnostic sensitivity and specificity [26].

Comparative analysis of FEVR subgroups revealed progressive anatomical and vascular changes associated with disease severity. As staging advanced, patients demonstrated significant reductions in multiple parameters: optic cup dimensions (horizontal and vertical diameters), optic disc horizontal diameter, all cup-disc ratios (area, horizontal, and vertical), and vascular metrics (VD, VL, AVD, and VFD). Conversely, the optic disc-to-macula distance showed progressive elongation. These findings suggest concurrent pathological processes - decreasing cup-disc ratios likely reflect optic nerve remodeling, while increasing disc-macula distance indicates posterior pole traction.

Our vascular parameter findings corroborate those of Koulisis et al. [27] using OCTA, who reported reduced VD and VFD in Stage 2 FEVR patients compared to Stage 1 cases. Their work linked diminished vascular branching complexity and VFD with late-phase angiographic posterior and peripheral vascular leakage (LAPPEL), a marker of vascular inflammation occurring when capillary loss exceeds neovascularization. This parallel suggests our observed vascular parameter reductions in Stage 2 patients may similarly relate to LAPPEL-mediated vascular changes. However, UWFFP-based validation studies are needed to confirm this potential association. Considering that VD did not show a significant difference between Stage 1 FEVR and the control group but significantly decreased in Stage 2 compared to both Stage 1 and the control group, VD may serve as an independent diagnostic marker for Stage 2 FEVR. This finding holds significance for monitoring disease progression.

Our findings specifically characterize retinal changes in uncomplicated Stage 1–2 FEVR cases through a comprehensive multimodal assessment incorporating optic disc/cup morphology, disc-to-macula distance, and retinal vascular parameters. This integrated approach minimizes diagnostic confusion with other retinal diseases that may share single-parameter similarities. Crucially, in clinical scenarios where FFA is contraindicated (e.g., pediatric patients or renal impairment), UWFFP with automated analysis provides a viable non-invasive alternative for initial assessment, demonstrating comparable diagnostic performance to FFA staging for key parameters like horizontal cup-disc ratio (AUC = 0.822) and vascular density (AUC = 0.77). While eliminating injection-related risks and reducing examination time by > 80% [6, 10], this approach maintains the caveat that FFA remains essential for detecting vascular leakage in Stage 3 + disease where UWFFP sensitivity declines. Collectively, these results establish that future FEVR diagnostic models should leverage multimodal parameter integration rather than single-metric reliance.

## Conclusions

This study quantifies 25 structural and vascular parameters from UWFFP using an automated EVisionAI platform, including previously unreported measurements in FEVR such as rim distance angles and optic cup roundness. By integrating structural and vascular biomarkers through a multimodal approach, we demonstrate UWFFP’s potential as a non-invasive alternative to FFA for initial evaluation in early stage, while establishing baseline parameters for future comparative studies. The analysis revealed significant differences in several parameters between FEVR and control groups, with horizontal cup-disc ratio, vertical cup-disc ratio, optic disc-to-macula distance, and VD demonstrating diagnostic potential. Notably, VD showed stage-dependent changes, suggesting particular value for identifying Stage 2 FEVR.

While this approach provides valuable insights into early disease manifestations, the current study’s clinical applicability is limited by both the exclusion of advanced-stage FEVR cases and the unvalidated specificity against other retinal disorders. Future research will expand to include more advanced FEVR cases while systematically evaluating diagnostic specificity against common retinal conditions such as ROP and high myopia. We will also focus on developing standardized protocols for longitudinal monitoring under a multicenter healthcare model, with the ultimate goal of establishing a robust framework for early FEVR detection and progression monitoring in clinical practice.

## Supplementary Information

Below is the link to the electronic supplementary material.

Supplementary Material 1

Supplementary Material 2

## Data Availability

No datasets were generated or analysed during the current study.
