# Supplementary material for: Retinal parameter analysis and diagnostic potential exploration in familial exudative vitreoretinopathy using ultra-widefield fundus photography
Source: Int J Retina Vitreous. 2025 Jul 30;11:87. doi: 10.1186/s40942-025-00716-y (PMC12312263; doi:10.1186/s40942-025-00716-y)
Supplement: Supplementary file 1 — Supplementary Material 1 [file 40942_2025_716_MOESM1_ESM.doc]

**Supplementary Table 1** Genetic characteristics and pathogenicity analysis of FEVR patients

| Gene | Transcript & Position | Nucleotide & Amino Acid Change | MAF | ACMG |
| --- | --- | --- | --- | --- |
| LRP5 | NM_002335.4: exon20 | c.4289delC: p.P1431Rfs*8 | - | LP |
| LRP5 | NM_002335.4:exon9 | c.2073G>T:p.p.W691C | - | LP |
| LRP5 | NM_002335:exon8 | c.1801G>A:p.G601R | 0. 0000083 | LP |
| LRP5 | NM_002335:exon6 | c.1283G>A:p.R428Q | 0.00005806 | LP |
| LRP5 | NM_002335:exon4 | c.728T>C:p.L243P | - | LP |
| LRP5 | NM_002335:exon2 | c.347delA:p.D116Afs*85 | - | LP |
| LRP5 | NM_001291902:exon23 | c.2933delC:p.S979Afs*7 | - | LP |
| LRP5 | NM_002335.4:exon2 | c.290C>T:p.A97V | 0.0034 | LP |
| LRP5 | NM_001291902.2:exon19 | c.2362_2363del:p.M788Vfs*2 | 0.000008264 | LP |
| LRP5 | NM_001291902.2:exon12 | c.1042G>A:p.A348T | 0.0001 | LP |
| LRP5 | NM_001291902.2:exon9 | c.107T>G:p.F36C | - | LP |
| LRP5 | NM_001291902.2:exon9 | c.253G>A:p.D85N | 0.0029 | LP |
| LRP5 | NM_002335.4:exon8 | c.1661delT:p.L554Rfs*35 | - | LP |
| LRP5 | NM_002335;exon5 | c.961T>C:p.C321R | - | LP |
| FZD4 | NM_012193.4:exon2 | c.1589G>A:p.G530E | 0.005 | LP |
| FZD4 | NM_012193.4:exon2 | c.1165G>A:p.G389R | 0.00002519 | LP |
| FZD4 | NM_012193:exon2 | c.313A>G:p.M105V | 0.00001666 | LP |
| FZD4 | NM_012193:exon2 | c.615_616insCA:p.Y206Hfs*35) | - | LP |
| FZD4 | NM_012193:exon1 | c.175A>G:p.N59D | - | LP |
| FZD4 | NM_012193.4:exon2 | c.686T>C:p.L229P | - | LP |
| FZD4 | NM_012193.4:exon2 | c.1511G>C:p.W504S | - | LP |
| FZD4 | NM_012193.4:exon1 | c.204delG:p.H69Tfs*11 | - | LP |
| FZD4 | NM_012193.4:exon2 | c.1589G>A:p.G530E | 0.005 | LP |
| FZD4 | NM_012193.4:exon1 | c.205C>T:p.H69Y | 0.001 | LP |
| TSPAN12 | NM_012338:exon8 | c.633T>A:p.Y211* | - | P |
| TSPAN12 | NM_012338:exon3 | c.149+1G>A | - | LP |
| TSPAN12 | NM_012338:exon6 | c.435G>A:p.W145* | - | LP |
| TSPAN12 | NM_012338:exon8 | c.633T>A:p.Y211* | - | P |
| TSPAN12 | NM_012338:exon4 | c.241G>A:p.G81R | - | LP |
| TSPAN12 | NM_012338:exon4 | c.241G>A:p.G81R | - | LP |
| JAG1 | NM_000214:exon26 | c.3391G>A:p.A1131T | 0.001 | LP |
| JAG1 | NM_000214:exon12 | c.1511A>G:p.N504S | 0.0006 | LP |
| JAG1 | NM_000214.3:exon7 | c.978G>C:p.E326D | - | LP |
| JAG1 | NM_000214.3:exon2 | c.133G>T:p.V45L | 0.0075 | LP |
| JAG1 | NM_000214.3:exon2 | c.133G>T:p.V45L | 0.0075 | LP |
| ILK | NM_001278442:exon12 | c.808-4G>A | 0.005 | VUS |
| ILK | NM_001278442:exon12 | c.808-4G>A | 0.005 | VUS |
| ILK | NM_001014795.3:exon2 | c.209C>G:p.P70R | - | LP |
| ILK | NM_001278441.2:exon11 | c.1168G>A:p.D390N | - | LP |
| EMC1 | NM_001271429:exon6 | c.626A>G:p.D209G | 0.0028 | VUS |
| EMC1 | NM_001271429.2:exon8 | c.914C>G | 0.001 | VUS |
| EMC1 | NM_001271429.2:exon18 | c.2219T>C:p.I740T | 0.000003181 | LP |
| ATOH7 | NM_145178:exon1 | c.455C>T:p.T152I | 0.001 | VUS |
| ATOH7 | NM_145178.4:exon1 | c.265A>G:p.I89V | - | LP |
| NDP | NM_000266:exon2 | c.110G>A:p.R37Q | 5.45584E-06 | LP |
| NDP | NM_000266:exon2 | c.110G>A:p.R37Q | 5.45584E-06 | LP |
| ZNF408 | NM_024741:exon5 | c.2117G>T:p.G706V | - | LP |

Explanations of the abbreviations in the table: FEVR: Familial Exudative Vitreoretinopathy; MAF: Minor allele frequency; ACMG: American College of Medical Genetics and Genomics; ACMG Classification: P (Pathogenic), LP (Likely Pathogenic), VUS (Variant of Uncertain Significance)

**Supplementary Table 2** Definitions and clinical significance of measured retinal parameters

Explanations regarding the units, symbols, abbreviations, and statistical parameters presented in the table: μm: micrometers; mm: millimeters; °: degrees.

| No. | Parameter (Unit) | Category | Definition | Clinical Significance |
| --- | --- | --- | --- | --- |
| 1 | Optic cup horizontal diameter (μm) | Optic Nerve Structure | Horizontal width of the optic cup | Evaluates cup morphology in naso-temporal dimension |
| 2 | Optic cup area (μm²) | Optic Nerve Structure | Surface area of the optic cup | Quantifies overall cup size and structural changes |
| 3 | Minimum disc-cup rim angle to horizontal line - end (°) | Neuroretinal Rim | Inferior rim angle relative to horizontal | Measures inferior neuroretinal rim configuration |
| 4 | Minimum disc-cup rim angle to horizontal line - start (°) | Neuroretinal Rim | Superior rim angle relative to horizontal | Measures superior neuroretinal rim configuration |
| 5 | Rim S distance (μm) | Neuroretinal Rim | Thickness of superior rim | Evaluates superior neural rim tissue |
| 6 | Rim N distance (μm) | Neuroretinal Rim | Thickness of nasal rim | Evaluates nasal neural rim tissue |
| 7 | Rim T distance (μm) | Neuroretinal Rim | Thickness of temporal rim | Evaluates temporal neural rim tissue |
| 8 | Optic disc-to-macula distance (μm) | Macular Topography | Distance between disc and macular centers | Assesses posterior pole anatomical relationships |
| 9 | Macula-optic disc line angle (°) | Macular Topography | Angle between macular-disc line and reference axis | Evaluates macular positioning |
| 10 | Optic disc short-to-long axis ratio | Geometric Feature | Ratio of shortest to longest disc axes | Evaluates disc ovality |
| 11 | Optic disc long-to-short axis ratio | Geometric Feature | Ratio of longest to shortest disc axes | Alternative measurement of disc ovality |
| 12 | Optic cup roundness | Geometric Feature | Circularity of optic cup (0-1) | Quantifies cup shape regularity |
| 13 | Vascular density | Vascular Metrics | Vessel area per unit retinal area | Measures retinal blood perfusion |
| 14 | Average vessel diameter (μm) | Vascular Metrics | Mean vascular caliber | Assesses vascular remodeling |
| 15 | Vessel length (mm) | Vascular Metrics | Total length of vascular network | Reflects vascular branching complexity |
| 16 | Vessel fractal dimension | Vascular Metrics | Branching pattern complexity (1-2) | Quantifies microvascular arborization |
| 17 | Optic cup vertical diameter (μm) | Optic Nerve Structure | Vertical height of the optic cup | Assesses cup morphology in superior-inferior dimension |
| 18 | Optic disc horizontal diameter (μm) | Optic Nerve Structure | Horizontal width of optic disc | Measures disc size in naso-temporal dimension |
| 19 | Optic disc elliptical short axis (μm) | Optic Nerve Structure | Short axis length of elliptical disc | Assesses disc morphology in its narrowest dimension |
| 20 | Rim I distance (μm) | Neuroretinal Rim | Thickness of inferior rim | Evaluates inferior neural rim tissue |
| 21 | Minimum disc-cup rim distance (μm) | Neuroretinal Rim | Shortest rim thickness | Identifies thinnest neuroretinal rim region |
| 22 | Area ratio of cup-disc | Structural Relationship | Ratio of cup area to disc area | Evaluates structural changes in optic nerve head |
| 23 | Horizontal cup-disc ratio | Structural Relationship | Horizontal cup-to-disc diameter ratio | Standard parameter for optic nerve assessment |
| 24 | Vertical cup-disc ratio | Structural Relationship | Vertical cup-to-disc diameter ratio | Important for glaucoma evaluation |
| 25 | Optic disc roundness | Geometric Feature | Circularity of optic disc (0-1) | Measures disc shape alterations |

Explanations regarding the units, symbols, abbreviations, and statistical parameters presented in the table: μm: micrometers; mm: millimeters; °: degrees.
