# Supplementary material for: Retinal parameter analysis and diagnostic potential exploration in familial exudative vitreoretinopathy using ultra-widefield fundus photography
Source: Int J Retina Vitreous. 2025 Jul 30;11:87. doi: 10.1186/s40942-025-00716-y (PMC12312263; doi:10.1186/s40942-025-00716-y)
Supplement: Supplementary file 2 — Supplementary Material 2 [file 40942_2025_716_MOESM2_ESM.docx]

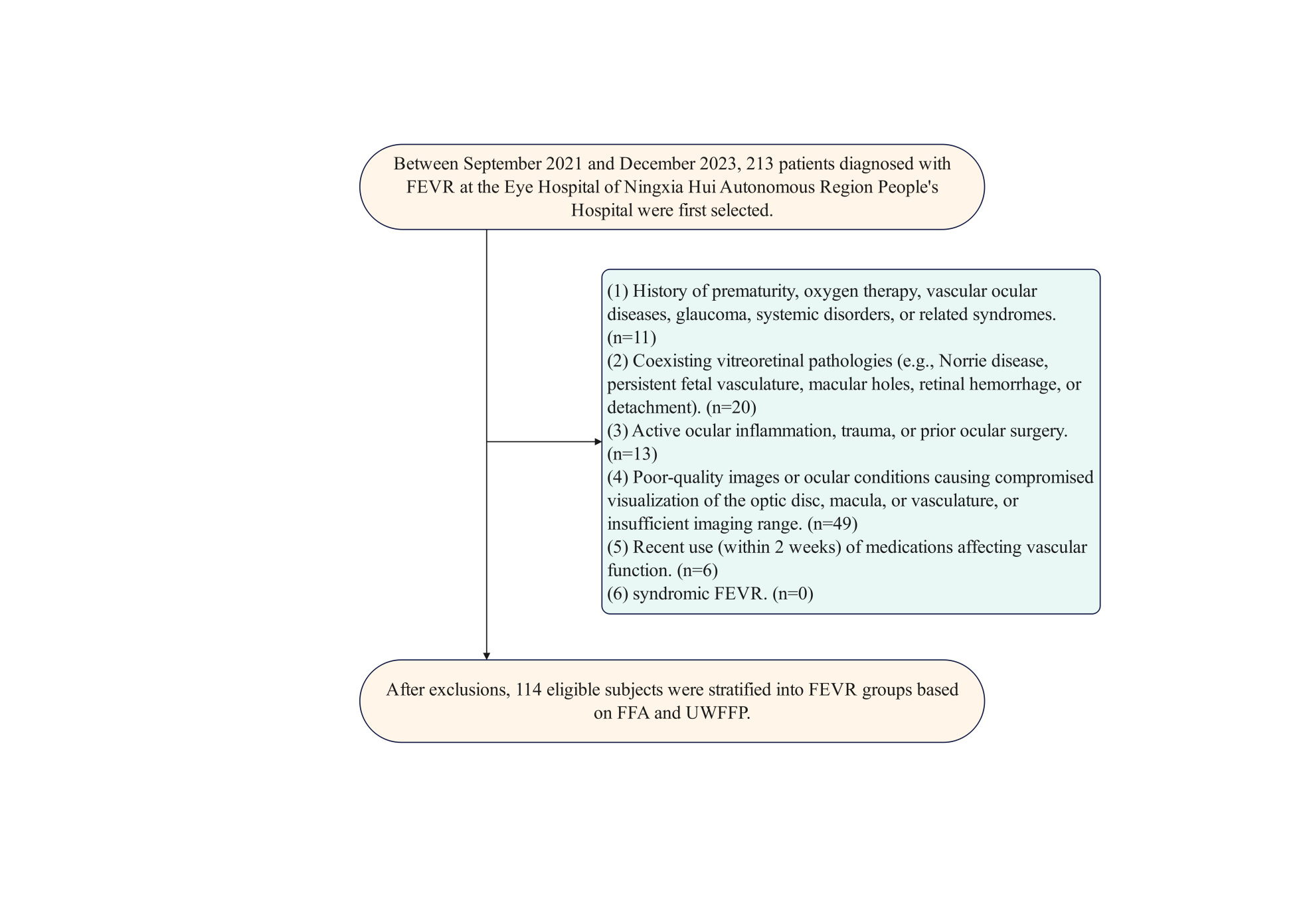


Fig. S1 Patient screening and enrollment flowchart. Of 213 initially identified FEVR patients, 114 met inclusion criteria and comprised the final study group.


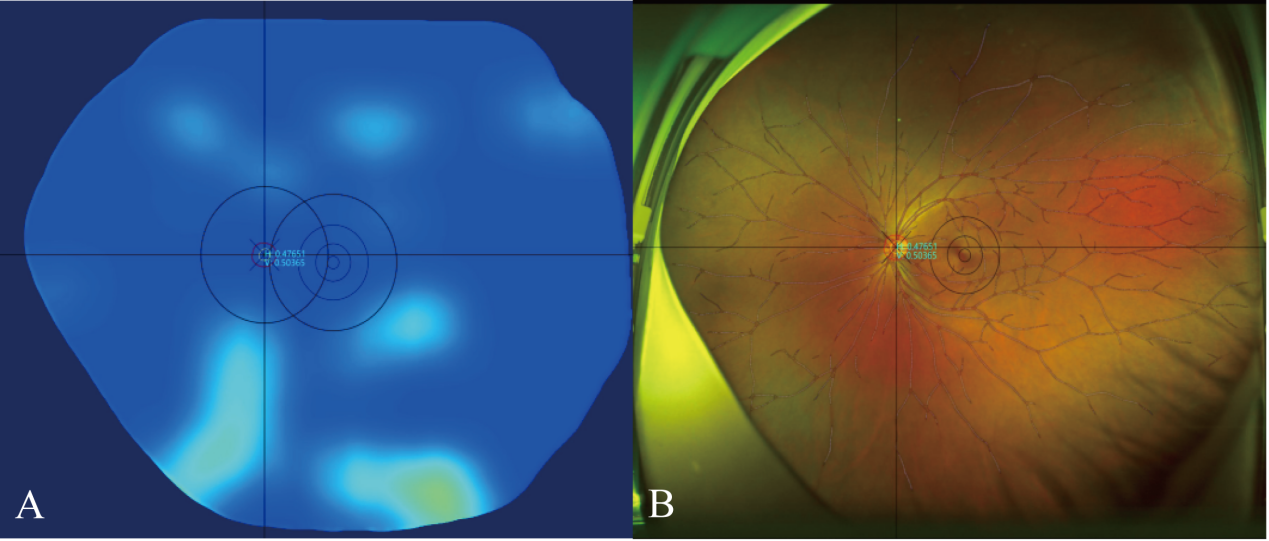


Fig. S2 Retinal images automatically segmented by the EVisionAI Cloud Platform. A Pixel area (Region of Interest, ROI) representing the retinal imaging field. B Automatic segmentation results of retinal structures and blood vessels.
